# Identification of genes controlling compatible and incompatible reactions of pearl millet (*Pennisetum glaucum*) against blast (*Magnaporthe grisea*) pathogen through RNA-Seq

**DOI:** 10.3389/fpls.2022.981295

**Published:** 2022-09-23

**Authors:** Shweta Singh, Rajan Sharma, Thirunavukkarasu Nepolean, Spurthi N. Nayak, Bheemavarapu Pushpavathi, Aamir W. Khan, Rakesh K. Srivastava, Rajeev K. Varshney

**Affiliations:** ^1^International Crops Research Institute for the Semi-Arid Tropics (ICRISAT), Patancheru, Telangana, India; ^2^Professor Jayashankar Telangana State Agricultural University (PJTSAU), Hyderabad, Telangana, India; ^3^ICAR-Indian Institute of Sugarcane Research, Lucknow, Uttar Pradesh, India; ^4^ICAR-Indian Institute of Millets Research, Hyderabad, Telangana, India; ^5^Department of Biotechnology, University of Agricultural Sciences, Dharwad, Karnataka, India

**Keywords:** differential reaction, blast, transcriptome, RNA-Seq, resistance

## Abstract

Blast [*Magnaporthe grisea* (Herbert) Barr] is an economically important disease in Asian pearl millet production ecologies. The recurrent occurrence of blast in the past one decade has caused enormous strain on grain and forage production. Identification of resistance genes is an important step to develop durable varieties. The present study is the first attempt to use RNA-Seq to investigate the transcript dynamics in a pearl millet inbred ICMB 93333, which had a unique differential reaction to two isolates—Pg 45 (avirulent) and Pg 174 (virulent) of *M. grisea.* The inbred was inoculated by both isolates and samples taken at six different time intervals for genome-wide RNA-Seq experiment. The transcriptome results revealed the differential expression of more than 2,300 genes. The time-specific comparison showed activation or repression of specific genes in various pathways. Genes and transcriptions factors related to pathogenesis-related proteins, reactive oxygen species generating and its scavenging genes, cell wall defense, primary and secondary metabolic pathways, and signaling pathways were identified by comparing the host-plant compatible and incompatible interactions. The genes identified from this experiment could be useful to understand the host-plant resistance and design novel strategies to manage blast disease in pearl millet.

## Introduction

Pearl millet [*Pennisetum glaucum* (L.) R. Br.], is the nutritious staple food for millions of poor people in the semi-arid tropics (SAT) region of the Indian sub-continent and many African countries (Martel et al., 1997; Rajaram et al., 2013; Vijayalakshmi et al., 2021). The crop is often jeopardized by various biotic and abiotic stresses, of which blast or leaf spot disease caused by the hemibiotrophic, ascomycete fungus *Magnaporthe grisea* (Herbert) Barr [anamorph: *Pyricularia grisea* (Cooke) Sacc.] has recently become a disease of economic concerns in India (Sharma et al., 2013). *Magnaporthe grisea* is known to affect both forage (Wilson and Gates, 1993) and grain pearl millet (Timper et al., 2002). The disease is characterized by grayish, water-soaked lesions which enlarge to become necrotic, resulting in extensive chlorosis and premature drying of young foliage (Singh et al., 2021). The disease initiates as lesions near leaf tips or margin or even both extending downwards on the outer edges (Wilson et al., 1989). The center of lesions is initially gray and water soaked which later turns brown surrounded by a yellow halo giving it the appearance of concentric rings (Kato, 2001).

The disease has increased at an alarming rate in the recent past, affecting most of the commercial single cross hybrids (Sharma et al., 2013). As of now, only a few pathotype-specific resistance sources such as ICMB 88004, ICMB 92444, ICMB 97222, ICMB 02111, ICMB 06444, ICMB 07111, ICMB 09333, ICMB 09999, ICMR 06222, ICMR 06444, ICMR 06111, ICMR 06666, and ICMR 11003 have been identified among designated hybrid parental lines of pearl millet, posing difficulties in disease management through host-plant resistance approach (Thakur et al., 2009; Goud et al., 2016; Singh et al., 2018). Pathogenic variability existing in the pathogen is another challenge in managing the disease through resistance breeding (Sharma et al., 2021). Hence, a better understanding of plant-pathogen interactions at the molecular level is essential to devise effective management strategies.

The plant-pathogen interactions have been studied through gene expression studies for important diseases in cereal crops such as wheat (Fofana et al., 2007), rice (Bagnaresi et al., 2012; Kawahara et al., 2012), sorghum (Yazawa et al., 2013), foxtail millet (Li et al., 2015) and pearl millet (Kulkarni et al., 2016). The recognition of pathogen-associated molecular patterns (PAMPs) by pattern recognition receptors (PRRs) on plant cell membrane is one such mechanism which results in the activation of pathogen triggered immunity (PTI; Jones and Dangl, 2006). The interaction of PRRs and PAMPs activates signal transduction cascade involving mitogen-activated protein kinase (MAPK), Ca^2+^, reactive oxygen species (ROS), virulence and transcription factors (Dangl and Jones, 2001; Macho and Zipfel, 2014). The activation of defense response induces cell wall strengthening, synthesis of phytoalexins, systemic acquired resistance (SAR), accumulation of signal molecules such as salicylic acid (SA), expression of pathogenesis-related (PR) genes and various other direct and indirect processes (Muthamilarasan and Prasad, 2013).

In the present study, RNA-Seq analysis was performed to investigate defense responses in incompatible and compatible interaction of a pearl millet genotype ICMB 93333 which shows differential reaction to two *M. grisea* isolates namely, Pg 45 from Patancheru, Hyderabad, and Pg 174 from Sambhal, Uttar Pradesh (Sharma et al., 2021). The study is aimed to identify key functional genes in resistant and susceptible responses and understand the molecular basis of incompatible and compatible interactions within a single genotype. Through the differential expression, novel genes and transcription factors involved in plant-pathogen interactions, especially defense-related responses, and primary and secondary metabolic pathways were identified that will help in breeding for blast resistance in pearl millet.

## Materials and methods

### Selection of plant material and pathogen isolates

The pearl millet inbred ICMB 93333, which showed differential reaction to the isolates Pg 45 (avirulent) and Pg 174 (virulent) of *M. grisea*, was selected for transcriptome profiling. The seeds of ICMB 93333 were sown in three pots of 15 cm diameter (15 seeds/pot), filled with sterilized soil-sand-FYM mix in the ratio of 2: 1: 1 and placed in greenhouse bay maintained at 30 ± 1°C at ICRISAT, Hyderabad in December, 2015. The isolates Pg 45 and Pg 174, were cultured on oatmeal agar (OMA) media for 7 days at 25 ± 1°C with 12 h of darkness. Spores were harvested by flooding the plates with sterile distilled water and the fungal growth containing mycelium and conidia was gently removed using a soft camel hair brush. The spore suspension was adjusted to the desired concentration (1 × 10^5^ spore ml^−1^) with the help of a haemocytometer and Tween 20 (polyoxyethylene sorbitan monolaurate) @ 0.02% vol/vol was added to the suspension prior to inoculation (Jia et al., 2003).

### Inoculation and sample collection

Pathogen inoculation and tissue harvest for RNA-Seq were performed as per the standardized protocols (Johnson et al., 2012; Sharma et al., 2013). The 12-day-old seedlings (Sharma et al., 2013) were selected for inoculation and sampling using a randomized design with three biological replicates for both isolates Pg 45 and Pg 174 during the early-morning hours. The seedlings were sampled at 0, 6, 12, 24, 48 and 72 h post inoculation (hpi). Samples from 10 to 12 seedlings/pot/replication per time point were collected in 50 ml Falcon tubes, immediately frozen in liquid nitrogen, and mixed to minimize the effect of transcriptome variability among individual plants. The harvested samples were stored at −80°C till further use.

### RNA extraction and library preperation

Total RNA was isolated from the samples by using XcelGen Plant RNA Mini Kit (Xcelris Genomics, India) according to manufacturer’s instructions. Quality and quantification were performed using Agilent Bioanalyser2100 (Agilent Technologies, Inc., Wilmington, DE, United States) and the Nano Drop (NanoDrop Technologies, Inc., Wilmington, DE, USA). RNA with integrity number (RIN > 7) was used for library preparation. Equal amounts of RNA from three biological replicates were pooled prior to cDNA preparation. The cDNA libraries were constructed, and paired end sequencing was carried out using an Illumina HiSeq TM 2500.

### Data analysis

The estimation of the quality of data generated per sample was done using Raspberry of NGS-QCbox (Katta et al., 2015). Further, Trimmomatic v0.35 (Bolger et al., 2014) was used to eliminate low-quality data. Precisely, (i) Adapter sequences from the reads were trimmed off (ii) the low-quality bases (PhredQ-score < 30) were removed, and (iii) the reads less than 30 bp in length were eliminated. The high quality reads of each sample thus generated were aligned to the pearl millet genome assembly (Varshney et al., 2017) using TopHat2 (Trapnell et al., 2009). Reads were assembled by Cufflinks v2.1.1 (Trapnell et al., 2010) using RABT method using minimum intron length as 20, fragment bias correction with rest default parameters was used. The obtained cufflink assemblies were then compared and merged using cuffmerge script from cufflinks to remove transfrags and generate a combined GTF for further downstream analysis. Differentially expressed genes (DEGs) were identified using Cuffdiff (Trapnell et al., 2012). A gene was considered as DEG if log2 fold change value was ≥+2 and ≤−2 (up-and down-regulation, respectively) and a *p*-value ≤ 0.05.

Heat maps were developed using MultiExperiment Viewer (MeV) v4.9. Transcription factors expressed in the current experiment were identified by BLASTX search against the plant transcription factor database (PlantTFdb). The annotation for the DEGs was determined using similarity of transcripts in different databases such as NCBI-NR, TrEMBL and Swiss-Prot databases with an E-value ≤ 1E−05. Pathway analysis was done using Blast2GO tool using Kyoto Encylcopedia of Genes and Genomes (KEGG) pathway database. The gene ontology (GO) classification was carried out using detailed gene information present in UniProtKB database. The GO terms enrichment and their interaction network was done using the BiNGO plugin in the Cytoscape tool. The redundancies of significantly enriched GO terms were reduced using REVIGO (similarity cutoff = 0.75).

## Results

### Data analysis

**Figure 1:**
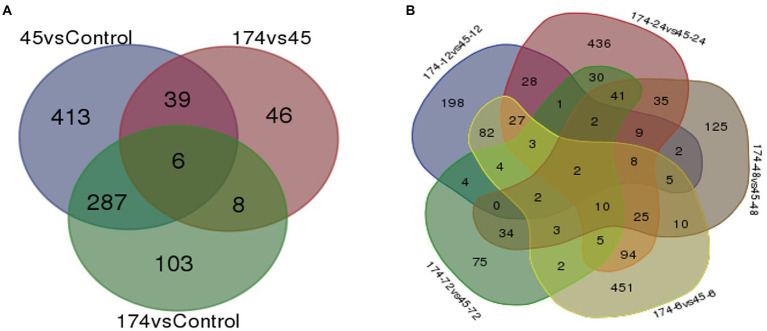
Uniquely and commonly expressed genes in pearl millet inbred ICMB 93333 during *M. grisea* infection by incompatible (Pg 45) and compatible (Pg 174) isolates **(A)** at all time periods and **(B)** at specific time-points.

The RNA-Seq generated a total of 1,103 million reads with an average of 92 million reads per sample. After stringent quality assessment and data curation, 983 million high quality filtered reads with an average of 82 million reads per sample were generated, in which 91 percent of the reads were aligned to the pearl millet genome (Table 1). The alignment of reads was observed on exons (58.9%–62.8%), introns (9.6%–11.0%) and intergenic regions (27.2%–30.6%). The relative abundance of reads in exonic, intronic, intergeneric and intronic/intergenic exon aligned regions is shown in Table 2. The number of differentially expressed transcripts in incompatible and compatible reaction of pearl millet genotype upon *M. grisea* inoculation at different time periods and across all time periods are shown in Figure 1.

**Table 1:** Basic statistics of the RNA-Seq experiment performed at six time-points in ICMB 93333 infected by two blast isolates.

Treatment	Reads generated	Reads after QC (>30%)	Total reads aligned	Alignment %
0 hpi (Control I)	90,495,394	85,284,710	75,134,209	88.09
Pg 45_6 hpi	168,230,688	146,793,563	135,289,293	92.16
Pg 45_12 hpi	134,811,778	117,452,939	110,224,633	93.84
Pg 45_24 hpi	130,104,806	112,964,339	104,688,750	92.67
Pg 45_48 hpi	155,590,416	136,795,380	125,636,039	91.84
Pg 45_72 hpi	60,443,492	57,391,057	48,795,678	85.02
Pg 174_6 hpi	54,997,350	52,321,973	45,777,930	87.49
Pg 174_12 hpi	69,530,998	61,248,867	56,895,884	92.89
Pg 174_24 hpi	53,368,586	47,231,778	44,165,318	93.50
Pg 174_48 hpi	71,286,978	62,232,330	57,852,539	92.96
Pg 174_72 hpi	62,788,224	54,820,397	49,667,413	90.60
72 hpi (Control II)	51,538,962	48,794,484	42,655,698	87.41
Total Reads	1,103,187,672	983,331,817	896,783,384	

The underlined terms indicate *M. grisea* isolate Pg 45 inoculated samples (incompatible interaction) at different time intervals.

**Table 2:** Mapping statistics of RNA-Seq reads aligned to different parts of the pearl millet genome during incompatible and compatible interactions to blast pathogen.

Treatment	Exonic regions	Intronic regions	Intergenic regions	Intronic/intergenic overlapping exon
0 hpi (Control I)	45,039,772 (62.8%)	7,121,156 (9.9%)	19,532,994 (27.2%)	11,476,688 (16.0%)
Pg 45_6 hpi	79,077,812 (60.5%)	14,372,133 (11.0%)	37,213,715 (28.5%)	19,117,325 (14.6%)
Pg 45_12 hpi	62,174,760 (59.3%)	11,575,949 (11.0%)	31,113,424 (29.7%)	15,498,518 (14.8%)
Pg 45_24 hpi	59,650,231 (58.9%)	10,536,301 (10.4%)	31,025,200 (30.6%)	15,668,142 (15.5%)
Pg 45_48 hpi	71,383,020 (59.1%)	12,625,529 (10.4%)	36,872,453 (30.5%)	18,785,137 (15.5%)
Pg 45_72 hpi	29,339,506 (62.7%)	4,486,541 (9.6%)	12,992,027 (27.8%)	7,234,742 (15.5%)
Pg 174_6 hpi	25,582,706 (59.7%)	4,708,785 (11.00%)	12,559,562 (29.3%)	6,757,105 (15.8%)
Pg 174_12 hpi	33,801,477 (61.9%)	5,940,010 (10.9%)	14,852,008 (27.2%)	7,692,027 (14.1%)
Pg 174_24 hpi	25,723,000 (62.6%)	4,171,713 (10.2%)	11,189,779 (27.2%)	5,994,875 (14.6%)
Pg 174_48 hpi	34,777,760 (62.5%)	5,492,722 (9.8%)	15,352,481 (27.6%)	8,267,108 (14.7%)
Pg 174_72 hpi	29,322,140 (62.2%)	4,709,761 (10.0%)	13,127,331 (27.8%)	6,715,039 (14.0%)
72 hpi (Control II)	24,910,997 (60.8%)	4,376,300 (10.7%)	11,715,804 (28.6%)	6,318,955 (15.4%)

The underlined terms indicate *M. grisea* isolate Pg 45 inoculated samples (incompatible interaction) at different time intervals.

### Functional annotation and classification of differentially expressed genes

**Figure 2:**
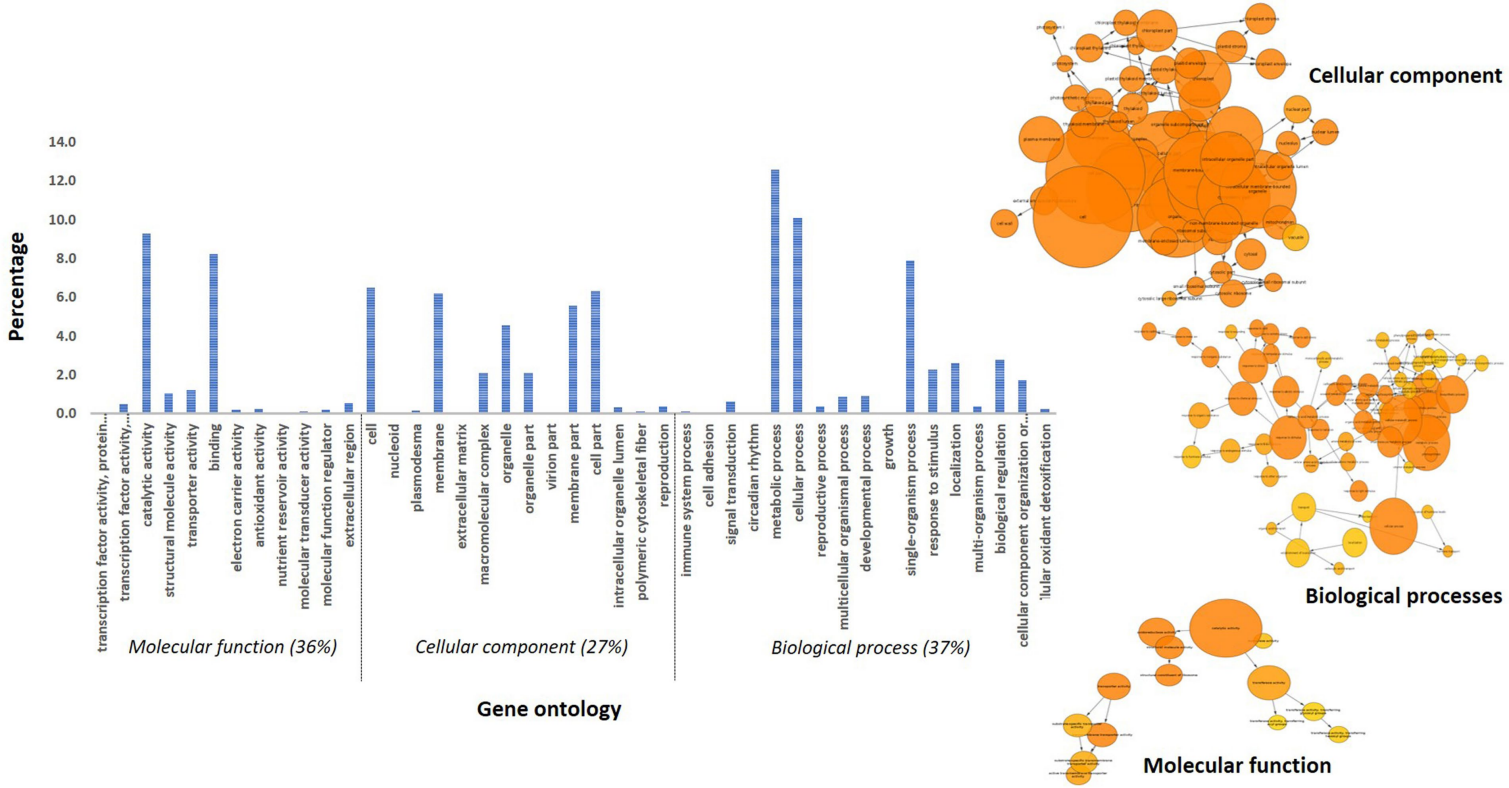
Gene ontology distribution of pearl millet transcripts and BINGO network categorized in cellular component, molecular function and biological process.

A total of 457 and 288 DEGs were up-and down-regulated, respectively, in incompatible reaction while 201 and 203 were induced and repressed, respectively in compatible reaction over the control samples. A total of 1,304 and 1,093 differentially expressed genes were up-and down-regulated, respectively. Among the control samples compared at 0 and 72 hpi, 692 genes were induced and 921 genes were repressed (Table 3). The identified functional classes of the DEGs were subjected to GO enrichment analysis. The number and assortment of the allocated GO categories provided a good indication of the diversity of the genes. According to the results of sequence alignments, differential sequences were classified into 43 functional groups, belonging to three main categories: cellular components, molecular functions and biological processes (Figure 2).

**Table 3:** Number of differentially expressed genes in *M. grisea* infected ICMB 93333 over different time-points.

Comparisons	Down-regulated genes	Up-regulated genes
Pg 174 vs. Pg 45:6 hpi	342	391
Pg 174 vs. Pg 45:12 hpi	173	204
Pg 174 vs. Pg 45:24 hpi	343	413
Pg 174 vs. Pg 45:48 hpi	64	249
Pg 174 vs. Pg 45:72 hpi	171	47
Control I vs. Control II	921	692
Pg174 all vs. Control all	203	201
Pg 45 all vs. Control all	288	457

**Figure 3:**
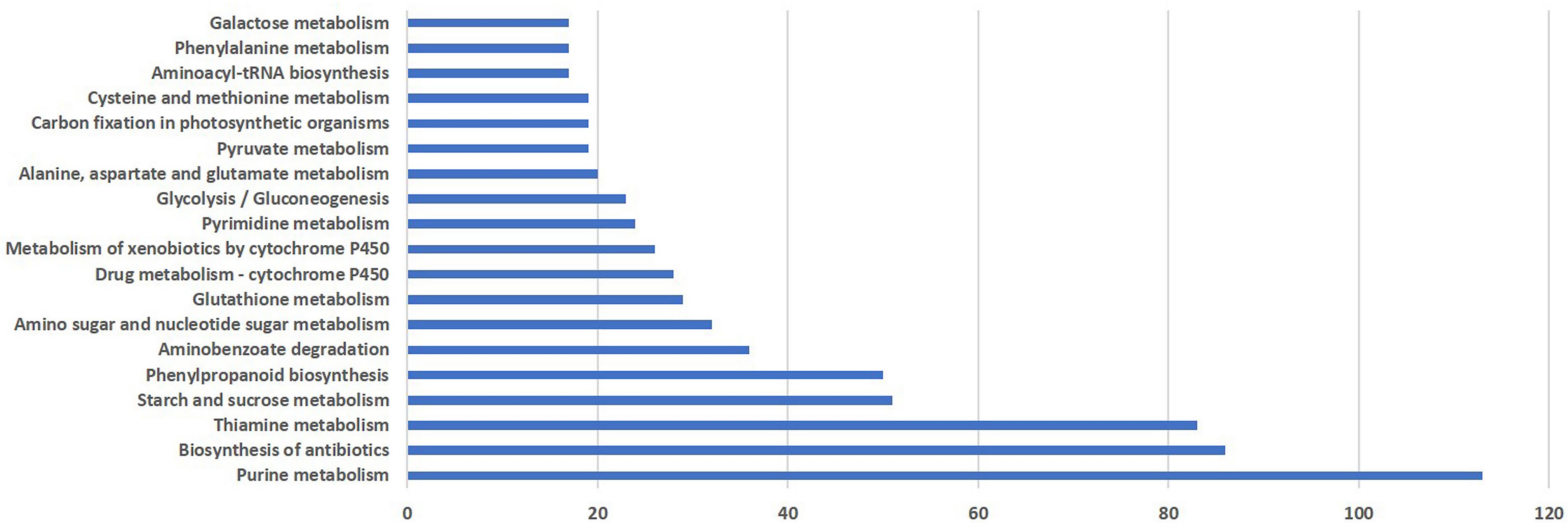
Distribution of pathways highly affected during *M. grisea* infection in pearl millet inbred ICMB 93333.

KEGG pathway analysis was performed to categorize the biological functions of DEGs. A total of 290 DEGs were allocated to 113 KEGG pathways. The pathways involving the highest number of DEGs was purine metabolism (113), followed by antibiotic biosynthesis (86), thiamine metabolism (81), starch and sucrose metabolism (51), phenylpropanoid pathway (50), amino benzoate degradation (36) and amino sugar and nucleotide sugar metabolism (32). Distribution of pathways that were highly affected during *M. grisea* infection is shown in Figure 3.

### Identification of defense-related genes in pearl millet in response to *Magnaporthe grisea*

**Figure 4:**
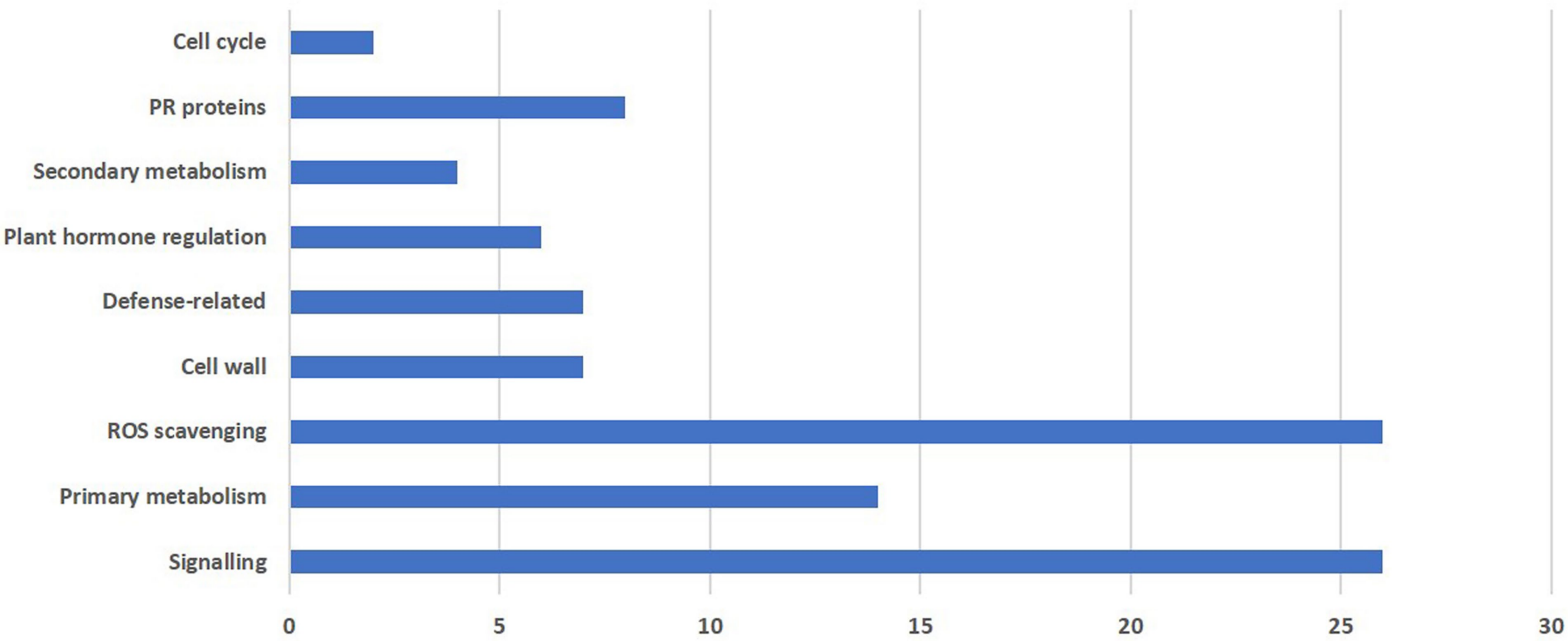
Functional grouping of total differentially expressed genes obtained from RNA-seq reads during *M. grisea* infection in pearl millet inbred ICMB 93333.

In the present study, differential expression was noticed for nine major groups as shown in Figure 4.

#### Pathogenesis related proteins

**Figure 5:**
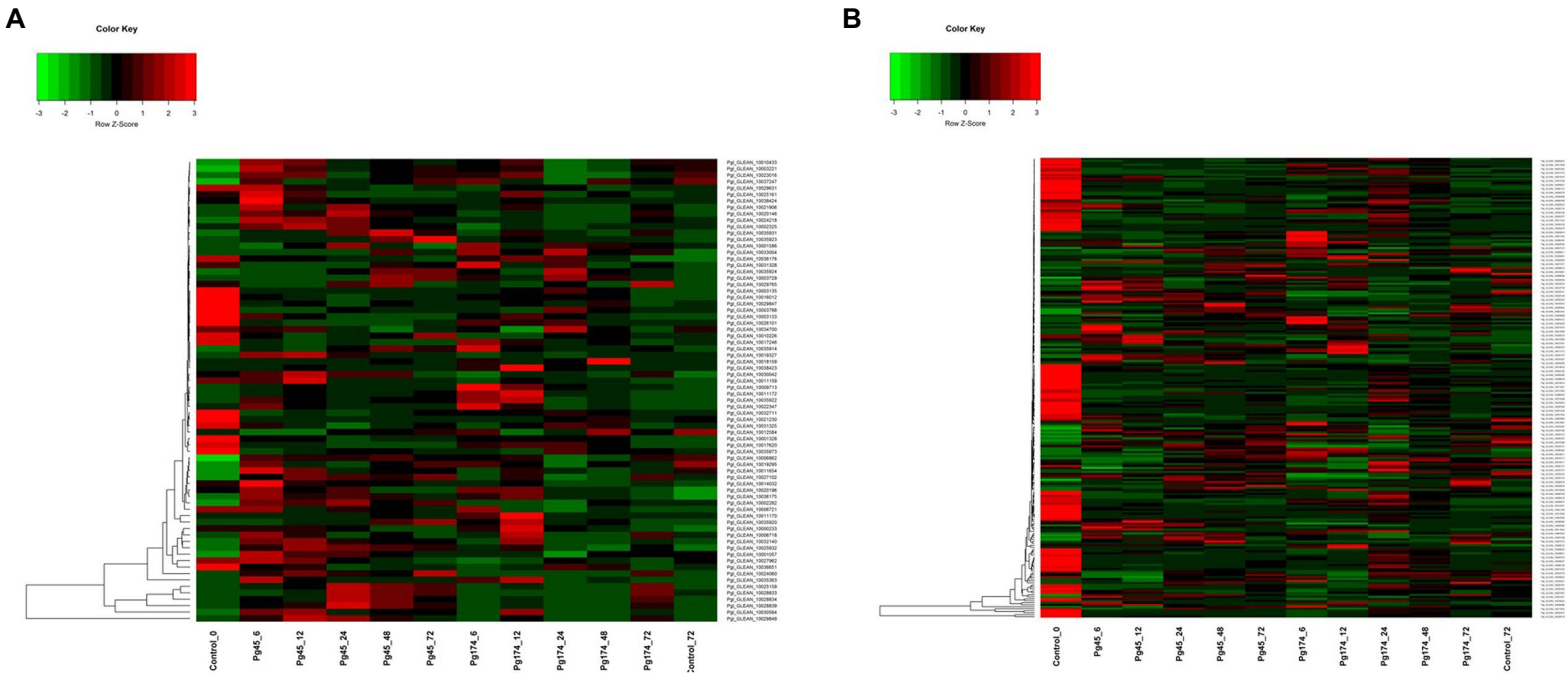
Heatmap representing relative levels of gene expression of **(A)** PR proteins and **(B)** ROS scavenging in ICMB 93333 when infected by Pg 45 and Pg 174 isolates.

A total of five different classes of PR proteins were differentially expressed in resistant (incompatible) and susceptible (compatible) interactions *viz.*, licheninases (β-1, 3-glucanases), chitinases and endochitinases, peroxidases, serine endopeptidase inhibitors (SEI) and ribonucleases. The number of differentially expressed transcripts for peroxidase of PR9, chitinase, β-1, 3-glucanases, ribonuclease and SEI family was 33, 16, 2, 15 and 6, respectively. The expression profiles of all the three genes were higher than control in both Pg 45 and Pg 174 inoculated plants (Figure 5A). Among these, the expression of peroxidase, licheninase, chitinase and SEI showed exponential increase in incompatible reaction at the initial stage of infection (6 and 12 hpi) with differential induction over other time intervals. Among the 15 transcripts of SPI, 11 transcripts were up-regulated in resistant reaction with 2.15-fold increase at 6 hpi in Pg45 inoculated sample (Pgl_GLEAN_10030564). The expression of chitinase gene increased in seven transcripts of Pg45 samples at 12 hpi with a maximum of 3.0-fold increase over Pg174 (Pgl_GLEAN_10002282). The expression of PR proteins was observed in different cellular components. Most of the serine protease inhibitors, peroxidase, chitinase, β-1, 3-glucanases and some ribonucleases were localized in extracellular membrane. An interesting observation in this study was the co-expression of β-1, 3-glucanases and chitinases in the resistant leaf samples. The licheninase Pgl_GLEAN_10029849 showed co-expression with chitinase and FPKM (3.37-fold) at 24 hpi in incompatible reaction while the expression remained higher than the compatible reaction at all time intervals.

#### Reactive oxygen species

The genes coding for ROS generating enzymes identified were peroxidase, phospholipases A_2_, xanthine dehydrogenase, amine oxidase, NADPH oxidase, proline dehydrogenase, and ROS scavenging enzymes were catalase, superoxide dismutase, alternative oxidase, glutathione transferase, aldehyde dehydrogenase, quinonereductase and ascorbate peroxidase. All these enzymes had higher expression than the control samples. Of a total of 33 transcripts for peroxidase, 18 were up-regulated while 15 were down-regulated in resistant reactions. Among these Pgl_GLEAN_10035363 had the maximum upregulation at 6 hpi in resistant reaction, 2.90-fold higher than the susceptible reaction (Figure 5B). A total of seven transcripts were expressed in cell wall with peroxidase having oxidoreductase activity and five transcripts were expressed in plasma membrane with NADPH oxidase activity. Most of the ROS generating transcripts *viz.* peroxidase, NADPH oxidase, xanthine dehydrogenase and amine oxidase were positively up-regulated until 48 hpi in incompatible compared to compatible response. In contrast, the ROS scavenging enzymes namely ascorbate peroxidases, catalase, alternative oxidase, glutathione S-transferase (GST), aldehyde dehydrogenase, superoxide dismutase and NADPH: quinonereductase showed repressed activities in initial stages of infection at 6 and 12 hpi but were up-regulated at later stages of interactions. The activity of catalase rose to maximum at 48 hpi in incompatible interaction with two times higher expression than the compatible interaction. Similarly, other ROS scavengers such as NADPH: quinone reductase, proline dehydrogenase and aldehyde dehydrogenase expressed higher at 72 hpi with 2-, 3- and 3-folds, respectively, over compatible interaction.

#### Alterations in the cell wall

**Figure 6:**
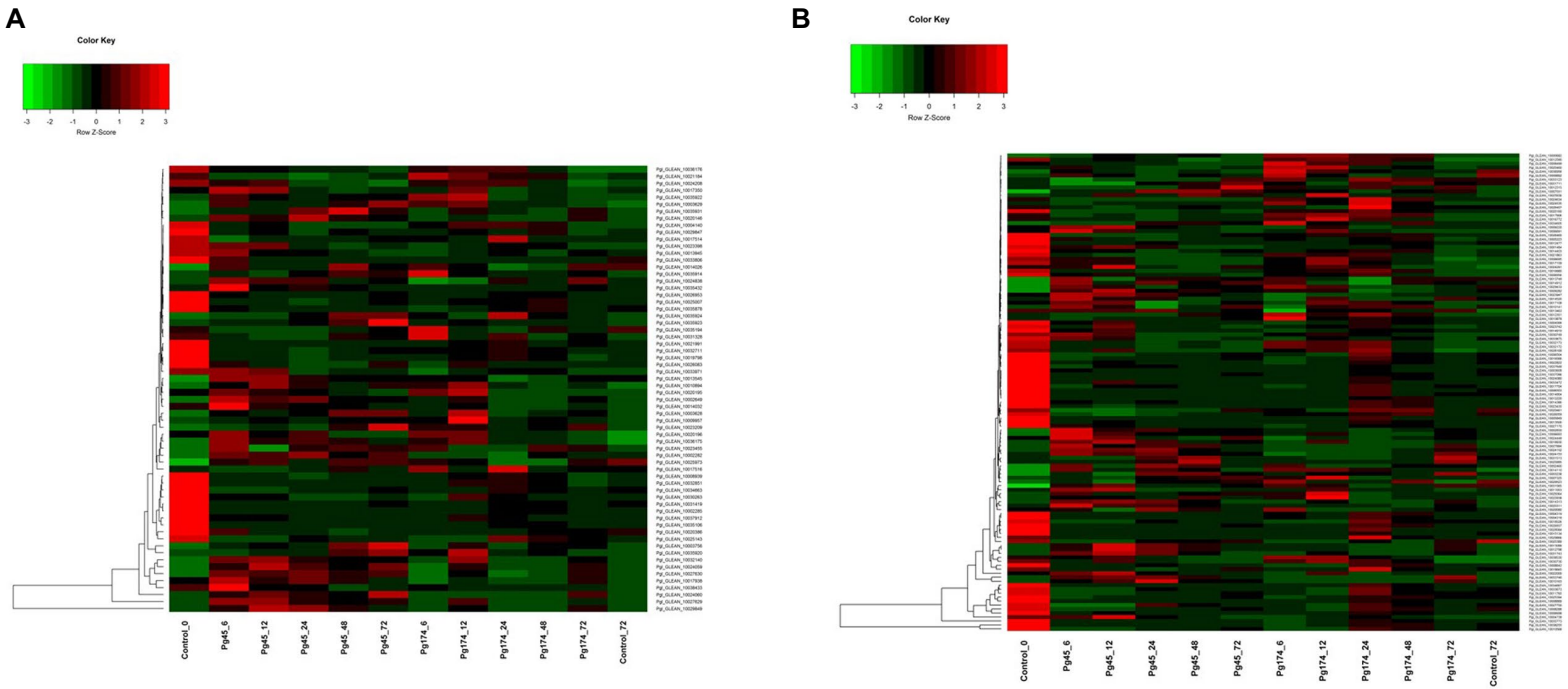
Heatmap representing relative levels of gene expression of **(A)** cell wall and **(B)** primary metabolism in ICMB 93333 when infected by Pg 45 and Pg 174 isolates.

The different plant cell wall strengthening enzymes were identified and found differentially expressed in the present study *viz.*, chitinase (16), cellulose synthase (10), cellulose (4), hydrolase hydrolysing O-glycosyl compounds (23), xyloglucan: xyloglucosyl transferase (6), Glucosylceramidase (1), Cinnamoyl CoA reductase (CCR) (1), Licheninase (1) and Lactoylglutathione lyase (1). There was up-regulation of cell wall and extra-cellular components, and the important genes related to strengthening of plant cell wall induced in response of pathogen attack namely cellulose synthase (GO: 0016760), cinnamoyl-CoA reductase (CCR; GO: 0016621), cellulose microfibril organization (GO: 0010215), licheninase, xyloglucan: xyloglucosyltransferase and lactoylglutathionelyase were highly up-regulated in incompatible interactions and were repressed in compatible interactions (Figure 6A). In incompatible interaction, the cellulose synthase along with cellulose microfibril organization activity was highly up-regulated. Six of the 10 transcripts for cellulose synthase were up-regulated in resistant response with transcript Pgl_GLEAN_10017350 being highly up-regulated (2.52-fold) at 6 hpi but declined after 12 hpi. The CCR was differentially expressed in both Pg45 and Pg174 inoculated seedlings. It was highly up-regulated in resistant reaction over all the time points of study except at 72 hpi with CCR transcript Pgl_GLEAN_10034663 showing maximum expression of 2.50-fold at 24 hpi. However, the expressions of cellulase, glucosylceramidase and CCR were also up-regulated in susceptible reaction.

There was also up-regulation of genes which coded for degradation of cell wall or mechanisms strengthening cell wall in compatible responses. The expression of lactoylgluthathionelyase, a component of cell wall protein was enhanced in susceptible reactions. The profile of this gene was much higher in Pg174 samples at 24 hpi. In the susceptible reaction, the activity of cellulase, the enzyme degrading the cellulose component of cell wall increased in all four transcripts from 6 hpi up to 24 hpi whereas its activity decreased at later stages. The highest activity was noted in Pgl_GLEAN_10008939 at 24 hpi (3.12-fold).

Induction of genes which coded for proteins degrading fungal cell wall was also expressed in the study. Two such genes expressing chitinase and glucosylceramidase were highly up-regulated in Pg 45 inoculated samples. Chitinase, causes breakdown of chitin into its monomer, thus disintegrating the fungal cell wall. In the present study, it was differentially expressed at all-time points, but showed several folds increase from 6 to 24 hpi in resistant interaction whereas the induction was quite slow in susceptible reaction. The expression of glucosylceramidase was down-regulated in both resistant and susceptible reactions; however, the susceptible reaction showed greater expression than resistant reaction. Glucosylceramidase, (Pgl_GLEAN_10021991) the enzyme catabolizing glucosylceramides, an important component of fungal plasma membrane, was highly suppressed (3.40-fold) at 24 hpi in compatible/susceptible reaction. In addition, the up-regulation of glycosyl hydrolase-encoding transcript was commonly observed.

#### Metabolic processes

The genes associated with metabolism of compounds such as amino acids, nitrogen, sulfur, nucleotides, phosphates, carbohydrates, lipid, fatty acids, vitamins, prosthetic groups and secondary metabolic processes were differentially expressed (Figure 6B). These genes formed the largest category, consisting of 1,388 genes among all biological processes. In the incompatible interaction, a high number of up-regulated genes were detected through 6, 12, 24, and 48 hpi, but several of them dropped off at 72 hpi. Expression profiles in compatible reaction also showed a great extent of variation during all time points. Some important metabolic processes affected during the pathogen infection have been described here:

#### Carbohydrate and energy

Genes involved in glycolysis and gluconeogenesis, electron transport and membrane-associated energy conservation, respiration, photosynthesis and other energy generation activities were included in this category. Phosphoglycerate kinase and hexokinase, the enzymes of glycolysis were up-regulated in resistant reaction with highest activity recorded at 6 hpi (2.02-and 4.09-fold, respectively). It is of interest that photosynthesis-related enzymes glyceraldehydes 3-phosphate dehydrogenase, fructose biphosphatealdolase and glycogen (starch) synthase were differentially expressed and suppressed in both compatible and incompatible interactions over control; however, the expression was much lower in resistant reaction (Figure 6B). The glyceraldehydes 3-phosphate dehydrogenase Pgl_GLEAN_10034667, fructose biphosphate aldolase Pgl_GLEAN_10008288 and glycogen (starch) synthase Pgl_GLEAN_10033673 had highest activity at 24 hpi in susceptible response (2.30-, 2.07- and 2.12-fold, respectively).

#### Protein and amino acid metabolism

Methylcrotonoyl-CoA-carboxylase (2) Pgl_GLEAN_10031743, glutamate dehydrogenase Pgl_GLEAN_10020311 (1) and anthranilate synthase (2) were the enzymes of amino acid metabolism differentially expressed but the former two were highly enhanced in resistant reaction at 6 and 12 hpi (Figure 6B). Asparagine synthase (2) transcript Pgl_GLEAN_10033746, another enzyme in amino acid synthesis was up-regulated in incompatible interaction with increase in activity from 6 to 24 hpi (5.24-fold). Sulfate adeylyltransferase, a pivotal enzyme for biosynthesis of sulfur containing amino acids cysteine and methionine was differentially expressed in both compatible and incompatible interaction whereas serine O-acetyltransferase another important enzyme for biosynthesis of cysteine and methionine was highly induced in incompatible interaction with increased expression at 24 hpi with highest fold change of 4.19 and 2.47 at 48 hpi. The gene encoding ubiquitin ligase was differentially expressed at all stages but was strongly induced at the initial stages of infection with a fold change of 2.27 (Pgl_GLEAN_10002650) at 6 hpi.

#### Lipid and fatty acid metabolism

The genes sustaining the fatty acid and lipid synthesis which were differentially expressed in this study included fatty-acyl-CoA reductase, linoleate 13S-lipoxygenase (LOX), those coding for lipid metabolism (GO: 006629), regulation of lipid metabolic process (GO: 0019216), fatty acid biosynthesis (GO: 0006633), beta oxidation of fatty acid (GO: 0006635), long chain fatty acyl metabolism (GO: 0035336; Figure 6B).

#### Secondary metabolism

**Figure 7:**
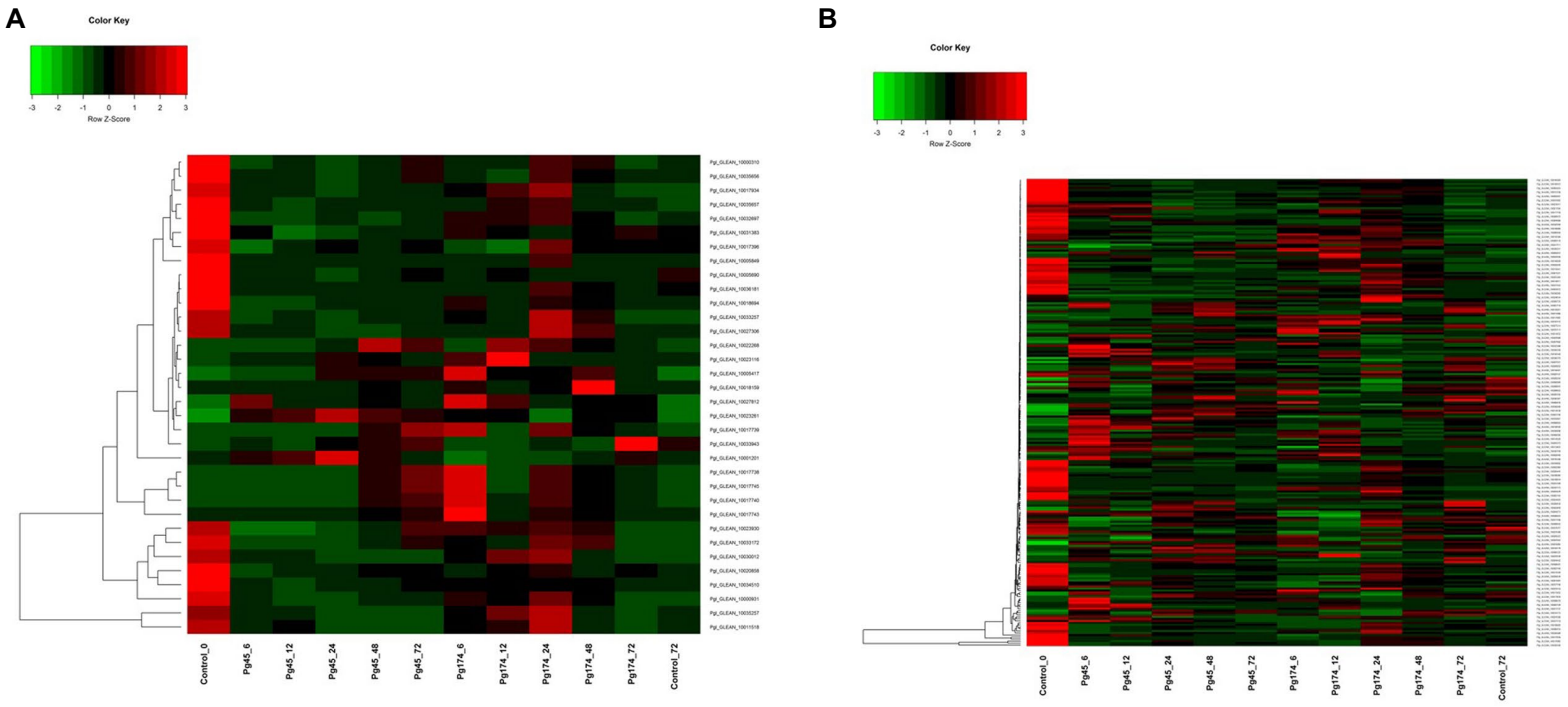
Heatmap representing relative levels of gene expression of **(A)** secondary metabolism and **(B)** signaling in ICMB 93333 when infected by Pg 45 and Pg 174 isolates.

In this study, we detected a number of genes for secondary metabolism related pathways which were enriched in up-regulated sets including biosynthesis of alkaloids from shikimates, purines, terpenoids and polyketides; phenylpropanoid pathway (23 transcripts) that encompassed those for flavonoid and lignin biosynthesis; mevalonate pathway (4), terpenoid pathway (5) and alkaloid pathway (1) (Figure 7A). A number of related pathways were enriched in up-regulated sets including biosynthesis of alkaloids from shikimates, purines, terpenoids and polyketides.

The extensively expressed genes of phenylpropanoid, flavonoid and shikimate pathway were naringenin chalcone, chalcone isomerase, 4-coumarate: CoA ligase, catechol-o-methyl transferase, phenylalanine ammonia lyase and prephenate dehydaratase. All of these were differentially expressed at all stages of infection, but the regulation was manifold in compatible and incompatible responses as compared to that of un-inoculated control. The naringenin chalcone synthesis was low in the initial stages of infection but increased at 48 hpi by 4 folds in incompatible interaction whereas in compatible stage the expression decreased at later stages.

### Cellular communication and signaling

The genes involved in signal transduction which were differentially expressed included calcium signaling pathway component calmodulin, ATPase, various classes of protein kinases (Figure 7B). An increase in the expression of these genes was observed in both interactions through all stages of study. The MAP cascade and MAPK induction were specifically higher (2.4-fold) in incompatible interaction at 12–24 hpi. However, there were differences in transcriptional levels of these groups of genes in resistant and susceptible responses. The maximum number of transcripts was attributed to serine protein threonine kinases (83) followed by receptor-like protein kinases (32). The genes for salicylic acid (SA) and jasmonic acid (JA) synthesis and regulation of defense pathways mediated by them were also observed at all time points. Interestingly, the biosynthesis of SA and JA were expressed together but the regulation of SA and JA were independent. The expression of ABC transporters and cytochrome p450 was also observed in this study.

### Transcription factors

**Figure 8:**
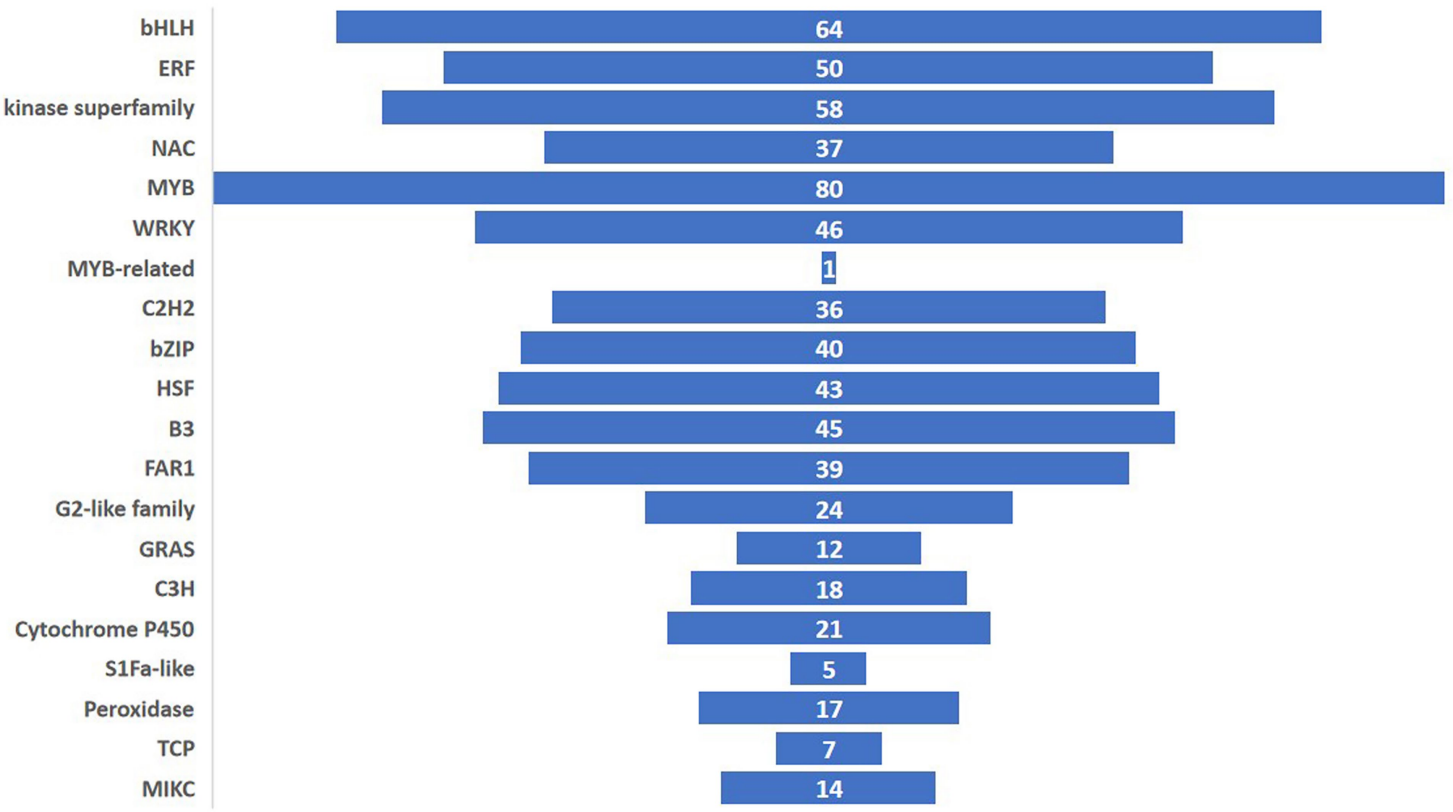
Number of pathogen-induced transcription factors for each family in ICMB 93333 at different time-points after inoculation with *M. grisea* isolates.

Among the 1,205 TFs, 20 different classes of TFs were induced of which the MYB family had the highest counts. The other important TFs induced in the experiment belonged to bHLH, ERF, kinase superfamily, WRKY, C2H2, bZIP, HSF, B3, FAR1, G2-like family, GRAS, C3H, TCP and MIKC (Figure 8). In this study, the highest expressed TF was MYB (93 genes) followed by bHLH (64 genes) and Kinase superfamily (58 genes). The transcription factor activity was annotated by the gene ontology GO: 0003700 throughout the study.

## Discussion

This is a first report which provides insights into compatible and incompatible reactions using single genotype of host pearl millet and two strains of the pathogen *M. grisea* through transcriptomic approaches. The compatible and incompatible interactions of pearl millet with *M. grisea* involved transcriptional activation or repression of a large number of genes and the pattern was consistent through different time points of infection. The fold-changes of the up-regulated genes were greater in the incompatible interaction even though there were significant commonly up-regulated genes in both interactions (Table 3). These observations supported the drastic infection-responsive reactions of pearl millet against blast fungus in the incompatible reaction. Similar studies of differential responses of rice defense-related transcripts to the infection of compatible and incompatible *M. grisea* strains were observed using RNA-Seq (Wang et al., 2009).

One of the features of plant defense response is the production of PR proteins that has been widely studied in plant pathogen resistance because they contribute to the increased resistance of the whole plant against a pathogenic attack (Ebrahim et al., 2011). The plant PR proteins are well characterized and have been classified into the PR-1 to -17 families (Sels et al., 2008). There were 15 up-regulated genes encoding for protease inhibitors belonging to the PR-1 family which is known to participate in the growth inhibition of pathogenic bacteria and fungi (Huang et al., 2005). The PR3, 4, 8, and 11 families are characterized as chitinases hydrolyzing chitin in cell walls of plant pathogens. The expression levels of 16 chitinases were up-regulated indicating that the degradation of the cell wall components (chitin) of pathogens is an important defense reaction in pearl millet against blast pathogen at the initial infection stage. The upregulation of these PR genes indicated that the degradation of the cell wall components (glucans, chitin and proteins) of pathogens protects the host against fungal pathogens at the initial infection stage. The extra-cellular secretion of chitinase in our experiment, suggested the degradation of the cell wall of pathogens and putative secretory nature of these proteins (Wang et al., 2009). The chitin binding and catabolic process associated with this PR protein is also indicative of *in-situ* cleavage of cell wall chitin polymers and macromolecules resulting in a weakened cell wall and rendering fungal cells osmotically sensitive (Huang et al., 2005). The PR protein β-1,3-glucanase known to hydrolyze β-1,3-glucans was also up-regulated as well as co-induced in this study. Such co-induction of these two proteins could effectively break the fungal cell wall and prevent further invasion of *M. grisea*. Similar results were reported in pea, soybean, and wheat (Boller, 1987; Mauch et al., 1988; Vogeli et al., 1988; Cheong et al., 2000).

The PR-9 family of peroxidases is also an oxidative enzyme which could be involved in defense reaction of pearl millet. Peroxidases form a complex family of proteins/ROS that catalyze the oxidoreduction of various substrates using H_2_O_2_. This class of PR protein shows maximum up-regulation in 33 transcripts in incompatible reaction. Peroxidase mediated H_2_O_2_ production causes oxidative burst which inhibits pathogen growth, signal induction of host defense responses and promotes hypersensitive response (O’Brien et al., 2012; Bigeard et al., 2015). Peroxidase along with laccase (LAC) utilizes H_2_O_2_ in the secondary cell wall and polymerises monolignols to lignins. This reaction leads to the formation of physical barrier at the cell wall to prevent pathogen penetration (Hatfield and Vermerris, 2001; Manjunatha et al., 2009). A higher and rapid accumulation of peroxidase in incompatible reaction of pearl millet during the infection of blast could lead to activation of such defense responses. The higher expression of peroxidase genes in the incompatible over compatible interaction was also reported earlier in pearl millet (Manjunatha et al., 2009), foxtail millet (Li et al., 2015), and rice against blast pathogen (Chittoor et al., 1997; Rauyaree et al., 2001; Wang et al., 2009).

Similar to peroxidases, the other ROS generating enzymes identified in our experiment were phospholipases A_2_, xanthine dehydrogenase, amine oxidase, NADPH oxidase and proline dehydrogenase. All the ROS related genes had higher expression in incompatible response than the control indicating their expression following recognition of fungal pathogen. On the other hand, enzymes namely catalase, alternative oxidase, glutathione S-transferase (GST), aldehyde dehydrogenase and NADPH: quinone reductase showed repressed activity at the initial stages (6 and 12 hpi) but increased at later stages of incompatible interactions. This can be supported by the fact that initial generation of H_2_O_2_ causes oxidative burst leading to hypersensitive responses and thus a manifestation of resistance but then overproduction of H_2_O_2_ induces the scavenging enzymes to manage the level of ROS generated.

The up-regulation of cellulose synthase plays an important role in defense by strengthening cell barriers and lignification (Vance et al., 1980), which indicates that strengthening physical barriers may be one of the factors for resistance in the inbred ICMB 93333 to *M. grisea* whereas a decline in the activity of cellulose synthase and cellulose microfibril in compatible interaction could be accounted for easy penetration by the pathogen and infection process. Another enzyme, cinnamoyl-CoA reductase (CCR) considered to play a significant role in cell wall re-inforcement through lignification and other defense mechanisms through activation of phytoalexins produced by monolignol synthetic pathways (Kawasaki et al., 2006), was differentially expressed in Pg45 and Pg174 inoculated seedlings. It was highly up-regulated in resistant reaction at all time points except at 72 hpi, since higher lignin synthesis is demanded at initial stages of host–pathogen interaction in resistant responses. The transcriptome profiling of sorghum and *Bipolaris sorghicola* (Yazawa et al., 2013) and barley powdery mildew interactions (Molitor et al., 2011) also showed a similar expression pattern of CCR in incompatible and compatible reactions. The expression of lactoyl gluthathionelyase, a component of cell wall protein was enhanced in susceptible reaction. It has an indirect involvement in initial defense process by degrading H_2_O_2_. The high expression in Pg174 inoculated samples at 24 hpi could be an indication of heavy degradation of H_2_O_2,_ thus making it unavailable for the polymerization of the cell wall and enabling easy penetration of pathogen. Cellulase, the enzyme degrading the cellulose component of cell wall, was also up-regulated in susceptible reaction and the expression increased 3-folds from 6 to 24 hpi. The cellulase expression in this study could be possibly a fungal transcript suggesting their active roles in degrading other plant cell wall components thus paving the path for easy penetration of the pathogen.

We found that the genes for photosynthesis related enzymes glyceraldehyde3-phosphate dehydrogenase, and fructose biphosphatealdolase and glycogen (starch) synthase were suppressed mainly in incompatible interaction suggesting that photosynthesis and assimilatory metabolism must be switched off to initiate respiration and other processes required for the defense (Scharte et al., 2005). On the contrary, the genes for protein and amino acids, fatty acids and lipids metabolisms were differentially expressed in both incompatible and compatible responses, but they were highly up-regulated in incompatible interaction. Most of the metabolic processes that lead to energy production such as glycolysis, pentose phosphate pathway, tricarboxylic acid cycle, electron transport chain, biosynthesis of ATP and some amino acids such as lysine and methionine whose catabolism leads to the production of energy are up-regulated under stress condition (Less et al., 2011).

The defense-associated TFs induced in this study included the members of the MYB families, WRKY, NAC, C2H2, bHLH, HSF, bZIP, ERF/AP2kinase superfamily, FAR1, G2-like family, GRAS, C3H, TCP and MIKC were reported to play crucial roles in plant defense (Singh et al., 2002; Buscaill and Rivas, 2014). MYB are widely used in the regulation of secondary metabolism in plants (Uimari and Strommer, 1997) as well as in disease resistance. The high expression of MYB was associated with JA and SA signaling pathways, and mediated response might lead to the over-expression of disease response in resistant/incompatible reaction (Mengiste et al., 2003). The involvement of MYB in defense responses by regulating SA and JA signaling was also reported in other crops like wheat and cotton (Cheng et al., 2016).

We identified 46 WRKY genes which regulate distinct groups of the plant defense genes mostly involved with immune responses. These proteins belong to a super-family of zinc finger proteins [WRKY-Glial Cell Missing (GCM1)] encoded in all higher plants. WRKY are implicated in plant defense responses, sugar signaling and chromatin remodeling. The expression of more than 70% of the *Arabidopsis* WRKY genes is influenced by various stresses, particularly by pathogen-related stimuli (Dong et al., 2003). OsWRKY13 enhances resistance in rice to the bacterial blight [*Xanthomonas oryzae* pv. *oryzae* (Xoo)] and the fungal blast (*M. grisea*; Qiu et al., 2007). Another class of TF induced was a bZIP protein (40 genes) which is linked to biotic stress which comprises TGA (TGACGTCA cis-element-binding proteins). TGA family members interact with an ankyrin repeat protein, non-expresser of PR genes (NPR1), which is a key component in the SA defense signaling pathway (Singh et al., 2002). The participation of bZIP in defense against *M. grisea* might have similar implications as that in rice (Okada et al., 2009).

A number of genes associated with intra-and inter-cellular communications, morphogenesis and receptor proteins were also differentially expressed in this study. The induction of protein kinases and MAP cascade genes at different times of infection supports involvement of these genes in defense response. The genes encoding for kinases and MAPKs cascades are all related with establishment of immune responses at particular stages during pathogen infection process. There was simultaneous but independent induction of genes for biosynthesis of SA and JA synthesis and regulation of defense pathways mediated by them. The KEGG pathway from our experiment revealed the high activation of thiamine metabolism pathways which could have played a greater role in enhanced resistance *via* up-regulation of PR genes and systemic acquired resistance mediated by salicylic acid production (Ahn et al., 2007). The accumulation of SA and JA in pathogen infection results in systemically acquired resistance (SAR) and is implicated in the activation of distinct sets of defense-related genes (Glazebrook et al., 2003). SA and JA mediated pathways identified in our experiment are known to cross-communicate with each other which induce a cascade of genes involved in signal transduction leading to enhanced defense responses.

The association of genes concerned with cell cycle, plant hormonal regulation, siroheme biosynthesis and transmembrane signal transduction were also identified. Among the phytohormone genes, the auxin and ethylene related genes were expressed in compatible and incompatible responses, respectively, and behaving antagonistic to each other. The expression of auxin-regulated proteins such as phosphoribosyl anthranilate isomerase and indole-3-glycerol-phosphate synthase was increased in susceptible whereas they were repressed in resistant reaction. Accumulation of more Indole 3-Acetic acid (IAA) in susceptible plants as compared to resistant plants was found during infection with different pathogens in rice (Fu et al., 2011). The role of auxin in disease development is associated with loosening of plant cell walls and depolymerization of wall polysaccharides, the first barrier against pathogens (Catala et al., 1997).

The aminocyclopropane-1-carboxylate synthase was induced in Pg45 inoculated seedlings at 6 hpi. The enhanced expression of the enzyme could have contributed for ethylene production from its precursor S-adenosylmethionine (SAM) which is produced from sterol adenosyl homocysteinase and 24-C-methyltransferase, the key enzymes of methionine pathway. It is well known that a large burst of ethylene is produced after the early stages of HR initiation and can induce plant defense-related pathways and genes (Ecker and Davis, 1987). Ethylene is involved in complex cross-talk of signaling pathways regulating plant defense responses to microbial attack. It acts in concert with signaling molecules, such as the antagonistic interactions described between SA and JA/ethylene (van Loon et al., 2006) or the synergistic action of SA and ethylene (Schenk et al., 2000). The significant up-regulation of the plant hormones in our experiment in incompatible reaction suggests their involvement in plant resistance response.

## Conclusion

The primary defense system in the host involved PR proteins and cell wall regulating genes which played the most important role in restricting the pathogen. The elevated expression of peroxidase, chitinase, β-1-3 glucanase, glucosylceramidase, cellulose synthase in the initial stage of infection continuing up to 24–48 hpi played a pivotal role in defense mechanism. The simultaneous upregulation of salicylic acid and jasmonic acid metabolism across the time intervals, the major players of signal transduction, also led to the induction of an array of defense responses. The information generated in this experiment could be valuable for pearl millet-blast interaction studies and the defense genes can serve as potential targets for genetic improvement of pearl millet with durable resistance. Future studies aim at comprehensive exploitation of pearl millet lines showing differential behavior toward the *M. grisea* pathotypes for identification of defense genes playing significant role in the host–pathogen interaction. The identified genes will be utilized for their expression profiles, sequenced and eventually used for mining functional SNPs which in the long run will pave way for development of durable varieties through accelerated breeding programs.

## Data availability statement

The data presented in the study are deposited in the NCBI SRA repository, accession number: https://www.ncbi.nlm.nih.gov/bioproject/PRJNA853892.

## Author contributions

RS, BP, SS, and RV conceived and designed the experiments. SS, SN, and RS performed the experiments. AK, SN, SS, TN, and RKS analyzed the data. SS, TN, RS, SN, RKS, RV, BP, and AK wrote the paper. All authors contributed to the article and approved the submitted version.

## Funding

This work was supported by the CGIAR Research Program on Grain Legumes and Dryland Cereals (CRP-GLDC) and Pearl Millet Hybrid Parents Research Consortium (PMHPRC).

## Conflict of interest

The authors declare that the research was conducted in the absence of any commercial or financial relationships that could be construed as a potential conflict of interest.

## Publisher’s note

All claims expressed in this article are solely those of the authors and do not necessarily represent those of their affiliated organizations, or those of the publisher, the editors and the reviewers. Any product that may be evaluated in this article, or claim that may be made by its manufacturer, is not guaranteed or endorsed by the publisher.
